# Distinct plasma metabolomic signatures differentiate autoimmune encephalitis from drug‐resistant epilepsy

**DOI:** 10.1002/acn3.52112

**Published:** 2024-06-21

**Authors:** Wenzheng Xiong, Tianrong Yeo, Jeanne Tan May May, Tor Demmers, Bryan Ceronie, Archana Ramesh, Ronan N. McGinty, Sophia Michael, Emma Torzillo, Arjune Sen, Daniel C. Anthony, Sarosh R. Irani, Fay Probert

**Affiliations:** ^1^ Department of Chemistry University of Oxford Oxford UK; ^2^ Department of Pharmacology, Medical Sciences Division University of Oxford Oxford UK; ^3^ Department of Neurology National Neuroscience Institute Singapore Singapore; ^4^ Duke‐NUS Medical School Singapore Singapore; ^5^ Lee Kong Chian School of Medicine Nanyang Technological University Singapore Singapore; ^6^ Nuffield Department of Clinical Neurosciences University of Oxford Oxford UK; ^7^ Department of Neurology John Radcliffe Hospital, Oxford University Hospitals Oxford UK; ^8^ Departments of Neurology and Neurosciences Mayo Clinic Jacksonville Florida USA

## Abstract

**Objective:**

Differentiating forms of autoimmune encephalitis (AE) from other causes of seizures helps expedite immunotherapies in AE patients and informs studies regarding their contrasting pathophysiology. We aimed to investigate whether and how Nuclear Magnetic Resonance (NMR)‐based metabolomics could differentiate AE from drug‐resistant epilepsy (DRE), and stratify AE subtypes.

**Methods:**

This study recruited 238 patients: 162 with DRE and 76 AE, including 27 with contactin‐associated protein‐like 2 (CASPR2), 29 with leucine‐rich glioma inactivated 1 (LGI1) and 20 with N‐methyl‐d‐aspartate receptor (NMDAR) antibodies. Plasma samples across the groups were analyzed using NMR spectroscopy and compared with multivariate statistical techniques, such as orthogonal partial least squares discriminant analysis (OPLS‐DA).

**Results:**

The OPLS‐DA model successfully distinguished AE from DRE patients with a high predictive accuracy of 87.0 ± 3.1% (87.9 ± 3.4% sensitivity and 86.3 ± 3.6% specificity). Further, pairwise OPLS‐DA models were able to stratify the three AE subtypes. Plasma metabolomic signatures of AE included decreased high‐density lipoprotein (HDL, −(C**H**_2_)_n_−, –C**H**_3_), phosphatidylcholine and albumin (lysyl moiety). AE subtype‐specific metabolomic signatures were also observed, with increased lactate in CASPR2, increased lactate, glucose, and decreased unsaturated fatty acids (UFA, –C**H**_2_CH=) in LGI1, and increased glycoprotein A (GlycA) in NMDAR‐antibody patients.

**Interpretation:**

This study presents the first non‐antibody‐based biomarker for differentiating DRE, AE and AE subtypes. These metabolomics signatures underscore the potential relevance of lipid metabolism and glucose regulation in these neurological disorders, offering a promising adjunct to facilitate the diagnosis and therapeutics.

## Introduction

Epilepsy is a heterogeneous neurological disorder characterized by recurrent and unpredictable epileptic seizures, affecting approximately 50 million people worldwide.^1^ Despite the availability of pharmacological treatments, a significant proportion of people with epilepsy (30%) experience drug‐resistant epilepsy (DRE) and do not respond to conventional therapies.^2^ Autoimmune encephalitis (AE) describes a group of autoantibody‐mediated brain disorders characterized by seizures and neuropsychiatric symptoms with autoantibodies targeting neuroglial cell‐surface proteins.^3^, ^4^, ^5^ AE typically gives rise to acute seizures which, like DRE, are often refractory to anti‐seizure medications (ASMs).^4^, ^5^ Further, many series in AE patients, especially those with leucine‐rich glioma inactivated 1 (LGI1)‐antibodies, identify cases originally diagnosed with a non‐autoimmune form of epilepsy.^6^, ^7^, ^8^ More rarely, acute AE gives rise to chronic epilepsy.^9^, ^10^

Timely diagnosis and initiation of immunotherapies are crucial for optimal prognosis in AE.^6^, ^11^ The diagnosis of AE typically involves a combination of clinical features, laboratory antibody tests and imaging.^6^, ^7^, ^8^, ^9^, ^10^, ^11^, ^12^ While the detection of neuronal surface antibodies (NSAbs) is a valuable tool, it can be expensive, laborious, and time‐sensitive, leading to potential delays in treatment initiation. Moreover, false positive antibody test results are well‐recognized to harm patient care^7^ and, as there are many seronegative cases, negative test results do not exclude AE.^13^ Hence, further adjunctive diagnostics are valuable to AE patients. They may also guide therapy and prognosis. Currently, no robust stratifying biomarkers exist.

Nuclear magnetic resonance (NMR) metabolomics, in combination with multivariate statistical techniques and machine learning, has emerged as a valuable approach for identification of potential biomarkers and disturbed metabolic pathways, as well as the diagnosis and staging of diseases.^14^ Recent studies have demonstrated the value of NMR metabolomics in detecting systemic inflammation and autoantibody‐mediated pathology in central nervous system (CNS) diseases with overlapping symptoms.^15^, ^16^ Previous work has demonstrated ^1^H NMR metabolomics can successfully discriminate between subsets of autoantibody‐mediated psychosis, distinguish multiple sclerosis from autoantibody‐mediated neuromyelitis optica spectrum disorder (NMOSD), and differentiate various subtypes of antibody‐mediated NMOSD.^15^, ^16^ In this study, we hypothesized that NMR metabolomics coupled with robust multivariate analytical methods might distinguish AE from DRE and, further, differentiate three of the commonest subtypes of AE, associated with autoantibodies against LGI1, N‐methyl‐D‐aspartate receptor (NMDAR) and contactin‐associated protein‐like 2 (CASPR2).

## Methods

### Human subjects

AE and DRE patients were recruited from John Radcliffe Hospital, Oxford, UK. The study was approved by the Research Ethics Committee (REC16/YH/0013) and all participants gave written informed consent. Matched clinical information was retrieved from the electronic patient record (Cerner Millenium). AE patients were diagnosed based on their clinical syndrome in association with serum and CSF antibody positivity at the peak of their disease determined by fixed and live cell‐based assays for CASPR2 and NMDAR‐antibodies, and serum positivity alone for LGI1‐antibodies, as described previously.^17^, ^18^ Inclusion criteria for DRE patients were stipulated such that: (1) DRE patients with known positive antibody results were excluded from the analysis, and (2) Patient records of the DRE patients were reviewed to further exclude cases potentially associated with autoimmune etiologies. Blood was collected in BD™ Vacutainer™ Lithium Heparin tubes (BD 367886) and plasma was isolated by centrifugation at 500 *g* for 10 min at room temperature prior to storage at −80°C.

### NMR spectroscopy

On the day of NMR data acquisition, plasma samples were defrosted at room temperature before being centrifuged at 100,000 *g* for 30 min at 4°C. 150 μL of the plasma samples were then mixed with 400 μL NMR buffer (75 mM phosphate buffer in D_2_O, pH 7.4) and transferred to a 5 mm borosilicate NMR tube (Norell).

NMR metabolomics analysis of plasma was conducted as previously described.^15^ NMR spectroscopy was performed using a 700‐MHz Bruker AVIII spectrometer (Department of Chemistry, University of Oxford) operating at 16.4 T equipped with a ^1^H [^13^C/^15^N] TCI cryoprobe at 298 K. ^1^H spectra of human plasma were acquired using a spin‐echo Carr–Purcell–Meiboom–Gill (CPMG) sequence (*τ* interval of 400 μs, 80 loops, 40 ms total filter time, 32 data collections, 1.5 s acquisition time, relaxation delay of 2 s, fixed receiver gain) to suppress broad signals arising from large molecular weight plasma components. For quality control, pooled samples were spread throughout the run to monitor technical variation.

Resulting free induction decays were zero‐filled by a factor of 2 and multiplied by an exponential function corresponding to 0.30 Hz line broadening prior to Fourier transformation. All spectra were phased, baseline corrected, and referenced to the lactate –CH_3_ doublet resonance at *δ* = 1.33 ppm, followed by visual inspection for errors and contaminations (Topspin 4.1, Bruker, Germany). Plasma NMR spectra were rationally divided into 122 spectral bins to avoid overlapping signals, integrated and normalized by the sum within each sample, accounting for any variations in sample dilution (ACD/Labs Spectrus Processor Academic Edition 12.01, Advanced Chemistry Development, Inc.). Integral values were pareto scaled prior to multivariate analysis.

Metabolite assignments for NMR signals was performed by referencing to literature values,^19^, ^20^, ^21^, ^22^ the Human Metabolome Database,^23^ and via 2D total correlation spectroscopy (TOCSY) experiments. Approximately 50 metabolites, including a range of lipoprotein and lipid species, amino acids, glucose, organic acids, nucleotides, and amides were identified.

### Statistical analysis

Multivariate analyses were performed in R software 4.1.2 (R Foundation for Statistical Computing, Vienna, Austria) using in‐house R scripts and the ropls package.^24^ Orthogonal partial least squares discriminant analysis (OPLS‐DA), a supervised method, was used to generate diagnostic models and identify significant differences in metabolite levels between groups. The number of orthogonal components was optimized through 10 repetitions of the default 7‐fold internal cross validation, with the final number determined by the median value obtained from the ten repetitions. OPLS‐DA models were validated using a 10‐fold external cross validation with 100 repetitions and permutation testing, as previously described.^16^ Details of model optimization and cross validation were described in Figure S1. Discriminatory variables were identified by calculating the average of the variable importance in projection (VIP) scores.

Univariate statistical analyses, such as Student's *t* test or one‐way ANOVA, were performed to identify significant differences in the mean for each discriminatory metabolite. Benjamini‐Hochberg method was used to control the false discovery rate at 0.05. Univariate Receiver Operating Characteristic (ROC) analyses and multivariate ROC analyses on a combination of features using logistic regression were performed using MetaboAnalyst 5.0.^25^ For patient demographic and clinical information, normality was tested by Anderson‐Darling test. Kruskal‐Wallis test with Dunn's multiple comparisons test was used to identify significant differences for non‐normal continuous variables. Chi‐Square test with Bonferroni correction for multiple comparisons was used for categorical variables. Adjusted two‐tailed *p*‐values ≤0.05 were considered statistically significant.

## Results

### Clinical features

The patient cohort (*n* = 238) comprised 162 DRE patients, and 76 AE patients including 27 with CASPR2‐, 29 with LGI1‐, and 20 with NMDAR‐antibody encephalitis. Baseline demographic and treatment details are summarized in Table 1. The median age of the DRE patients was 37 years old and 62% were female. As expected, CASPR2 and LGI1 patients were older compared to DRE and more were males (89% and 79%, respectively), whereas NMDAR‐antibody encephalitis patients had a median age of 30 and were predominantly female (95%).^6^, ^17^, ^18^ While all DRE patients were receiving ASMs (100%), the percentage was lower in AE patients (54%) who frequently received immunotherapies. Again, as expected, more AE patients (19% CASPR2‐, 24% LGI1‐, 40% NMDAR‐antibody patients) had systemic tumors, also focal and generalized seizures contrasted across the cohorts. DRE patients were relatively stable and provided their blood samples during routine outpatient clinics, while AE patients were potentially sampled both during acute in‐patient stays and at outpatient clinics.

**Table 1:** Patient demographic and clinical information.

	DRE (*N* = 162)	AE‐CASPR2 (*N* = 27)	AE‐LGI1 (*N* = 29)	AE‐NMDAR (*N* = 20)	*p* value (adjusted *p* value)
Age, median (IQR)	37 (27, 48)^C L^	74 (66, 78)^D N^	72 (57, 73)^D N^	30 (23, 58)^C L^	<0.001 (<0.001)
Sex, *n* (%)					
Female	100 (62%)^C L N^	3 (11%)^D N^	6 (21%)^D N^	19 (95%)^C D L^	<0.001 (<0.001)
BMI, median (IQR)	27 (24, 31)	27 (23, 28)	26 (20, 31)	28 (21, 32)	0.7 (>0.9)
Unknown	60 (37%)	20 (74%)	21 (72%)	10 (50%)	
Use of ASMs, *n* (%)					<0.001 (<0.001)
Yes	162 (100%)^C L N^	17 (63%)^D N^	20 (69%)^D N^	4 (20%)^C D L^	
Unknown	0	3 (11%)	2 (7%)	0	
Use of steroids, *n* (%)					<0.001 (<0.001)
Yes	1 (1%)^e^ ^,^^C L N^	6 (22%)^D L^	17 (59%)^C D^	7 (35%)^D^	
Unknown	0	1 (4%)	2 (7%)	1 (5%)	
Use of other immunotherapies, *n* (%)					<0.001 (<0.001)
Yes	2 (1%)^C L N^	9 (33%)^D^	11 (38%)^D^	9 (45%)^D^	
Unknown	0	1 (4%)	2 (7%)	0	
Identified tumor(s), *n* (%)^a^					0.0013 (0.010)
Yes	21 (13%)^L N^	5 (19%)	7 (24%)^D^	8 (40%)^D^	
Unknown	0	7 (26%)	12 (41%)	0	
Seizure semiology, *n* (%)					<0.001 (<0.001)
Focal seizures	121 (75%)^L N^	19 (70%)	24 (83%)^D^	1 (5%)^D^	
Focal^b^	65	19	20	1	
Focal + FBTCS^c^	56	0	4	0	
Generalized	39 (24%)^L N^	1 (4%)	1 (3%)^D^	5 (25%)^D^	
GTCS	36	1	1	5	
Other^d^	3	0	0	0	
Unknown	2 (1%)	7 (26%)	4 (14%)	14 (70%)	
Disease duration (from onset to sampling date, months), median (IQR)	160 (180)^C L N^	38 (38)^D^	25 (46)^D^	16 (24)^D^	<0.001 (<0.001)
Unknown	1 (1%)	0	0	0	
Seizure‐free days (from last seizure to sampling date), median (IQR)	16 (143)^C L N^	646 (1369)^D^	272 (726)^D^	814 (761)^D^	<0.001 (<0.001)
Never had seizures	0	7 (26%)	2 (7%)	12 (60%)	
Unknown	0	2 (7%)	3 (10%)	2 (10%)	

Kruskal‐Wallis test with Dunn's multiple comparisons test was used to identify significant differences of each class in age, BMI, disease duration and seizure‐free days. Pairwise Chi‐Square test with Bonferroni correction for multiple comparisons were used for categorical variables. Omnibus *p*‐values and adjusted omnibus *p*‐values with Bonferroni correction across demographic variables were reported. D, C, L, and N indicate a significant difference (*p* < 0.05) exists with DRE, CASPR2, LGI1, NMDAR, respectively, in the corresponding post‐hoc multiple comparisons.

GTCS, generalized tonic–clonic seizure; IQR, interquartile range.

^a^ Identified tumors encompass any tumor (including cancer) detected anywhere in the whole body (including brain), as documented in the electronic patient records at the time of blood sampling.

^b^ Includes focal aware seizures and focal impaired awareness seizures.

^c^ Focal seizures and focal to bilateral tonic–clonic seizures (FBTCS).

^d^ Absence seizures, myoclonus.

^e^ The patient was on lifelong hydrocortisone replacement due to childhood‐onset hypopituitarism, unrelated to autoimmune pathology.

### NMR plasma metabolomics coupled with OPLS‐DA models discriminate autoimmune encephalitis patients from those with drug‐resistant epilepsy

**Figure 1:**
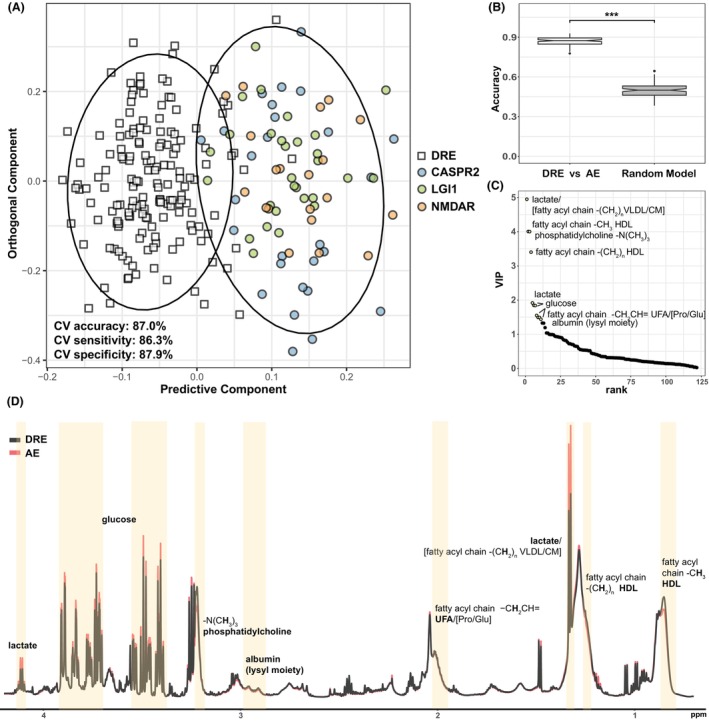
Altered plasma metabolome between AE patients and DRE patients. (A) Representative OPLS‐DA scores plot showing separation of AE (circle, *n* = 76) from DRE (square, *n* = 162) patient plasma samples. AE plasma samples are colored according to the subtype (autoantibody specificity, CASPR2/LGI1/NMDAR, blue/green/orange). The ellipses indicate 95% confidence interval. The OPLS‐DA model was generated with 1 predictive component and 7 orthogonal components. CV, cross validation. (B) Predictive accuracy of the ensemble of the OPLS‐DA models compared with that of the randomly permutated null distribution. Kolmogorov–Smirnov test. ***p <0.001. (C) Discriminatory metabolites driving the separation of the OPLS‐DA models, ranked by VIP scores. The top 11 resonances, identified with the inflexion point with a VIP score cutoff of 1.4, were labelled. “/” indicates the mentioned metabolites are overlapped in the spectral region. Metabolite names in square brackets refers to non‐dominant overlapping metabolites also found in that spectral region. VLDL, very‐low‐density lipoproteins. CM, chylomicrons. (D) Mean NMR spectra of plasma samples from AE (red, *n* = 76) and DRE patients (black, *n* = 162) highlighted and labelled with discriminatory metabolites derived from the OPLS‐DA models.

To compare plasma metabolomic signatures between DRE (*n* = 162) and AE patients (*n* = 76), ^1^H NMR spectroscopy was performed with predictive models of OPLS‐DA using 10‐fold external cross validation. Cross validation and permutation testing showed that the model was able to identify AE patients in the test set from DRE patients with 87.0 ± 3.1% accuracy, 87.9 ± 3.4% sensitivity and 86.3 ± 3.6% specificity and the model performed significantly better than random chance (50.0 ± 5.3% accuracy, 50.0 ± 6.9% sensitivity, 49.8 ± 7.4% specificity, *p* <0.001, Kolmogorov–Smirnov test), indicating it is both robust and not a result of overfitting (Fig. 1A–C, Table S1). In addition, NMR spectra were also obtained for three subjects selected to have post‐AE epilepsy who had AE for 2–3 years before being treated as epilepsy with only ASMs (refer to Table S2 for detailed case information). Notably, when applying this OPLS‐DA model to predict these three patients using their plasma metabolome, all three patients were classified as epilepsy, clustered with the DRE group (Fig. S2).

Mean spectra from DRE patients and AE patients (Fig. 1D) show discriminatory metabolites derived from the model. Compared to DRE patients, AE patients had increased plasma lactate, glucose and decreased high‐density lipoprotein (HDL, fatty acyl chain –(C**H**_2_)_n_–, –C**H**_3_ in lipoproteins, the spectral integral predominated by HDL), phosphatidylcholine (N^+^(C**H**_3_)_3_, choline‐containing phospholipids, predominantly phosphatidylcholine), unsaturated fatty acids (UFAs, –C**H**_2_CH = from the unsaturated fatty acyl components) and albumin (lysyl moiety of albumin)^22^ (Table S3).

**Figure 2:**
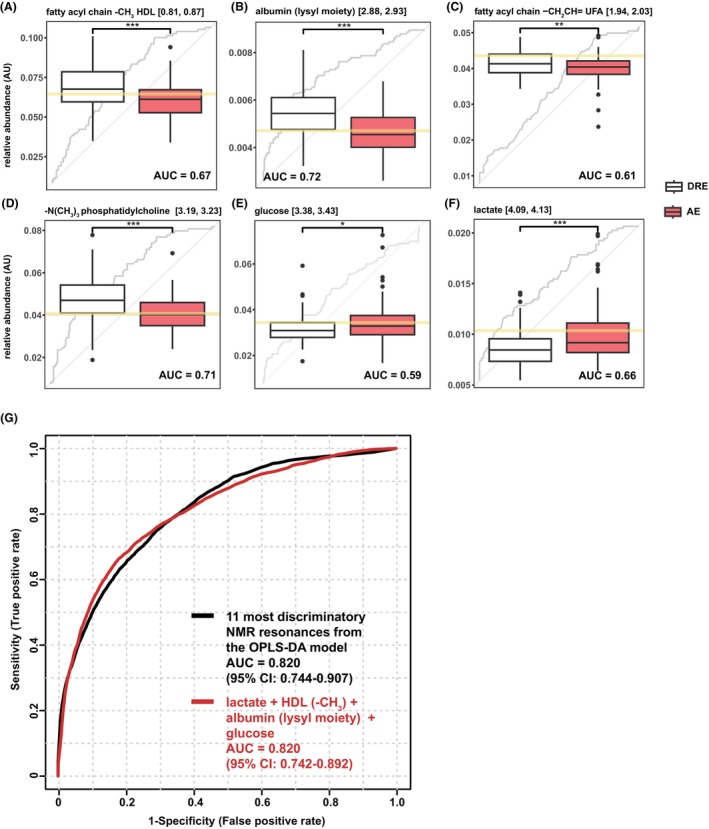
ROC analysis for discriminatory metabolites. (A–F) Boxplots of the highest ranked discriminatory metabolites identified by the OPLS‐DA model in AE versus DRE. Gray lines were ROC curves of each metabolite. Yellow lines indicate optimal cutoff (closest to top‐left corner) from univariate ROC analyses. (G) Multivariate ROC analysis on a combination of 11 most discriminatory NMR resonances from the OPLS‐DA model (black) and on 4 selected features (red).

Univariate ROC analysis was conducted for each of the most discriminatory metabolites, indicating their individual potential to classify AE and DRE patients with an AUC ranging from 0.59 to 0.72 (Fig. 2A–F). Multivariate ROC analysis coupled with logistic regression on all the 11 most discriminatory resonances yielded an AUC of 0.820 (95% CI: 0.744–0.907). Notably, when selecting lactate, HDL (–C**H**_3_), the albumin lysyl moiety, and glucose, four features with lower covariation that are routinely measurable in the clinical setting, the ROC analysis showed a comparable AUC of 0.820 (95% CI: 0.742–0.892) (Fig. 2G).

### Distinct metabolomic signatures identified for each AE subtype

**Figure 3:**
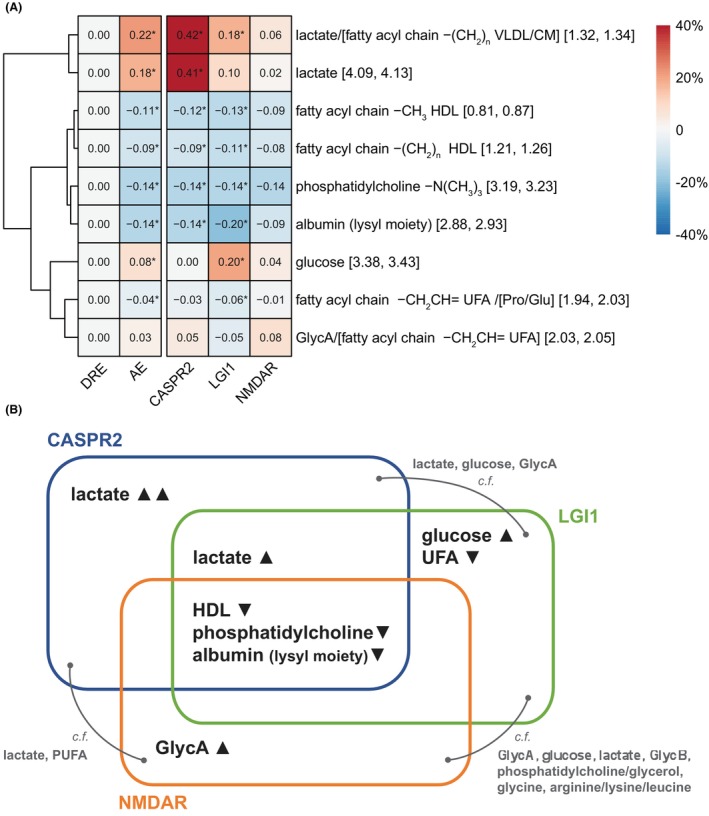
Specific alteration in plasma metabolome in each AE subtype. (A) Heatmap of percentage changes in key metabolites identified by the OPLS‐DA models of AE versus DRE, and in each AE subtype relative to the DRE group. Numbers in the square brackets represent the boundary of corresponding spectral region in ppm. “/” indicates the mentioned metabolites are overlapped in the spectral region. Metabolite names in square brackets refers to non‐dominant overlapping metabolites also found in that spectral region. * Significance in mean compared to DRE group (*q* < 0.05 in univariate analysis). (B) Venn diagram illustrating metabolic signatures of AE subtypes. Metabolites in black were identified from OPLS‐DA models of AE versus DRE, and each AE subtype versus DRE, while metabolites in gray were identified from OPLS‐DA models of pairwise AE subtype comparisons. HDL, high‐density lipoprotein. UFA, unsaturated fatty acids. PUFA, polyunsaturated fatty acids. GlycA/B, Glycoprotein A/B.

Upon further examination of the discriminatory metabolites, each AE subtype appeared to have its own unique metabolic signature apart from the shared metabolomic perturbation in HDL –(C**H**_2_)_n_–, HDL –C**H**_3_, phosphatidylcholine and the albumin lysyl moiety (Fig. 3A). Plasma lactate levels were increased in LGI1‐antibody encephalitis patients, and, even more so, in CASPR2‐antibody encephalitis patients. Elevated plasma glucose and decreased UFA were only observed in the plasma of LGI1‐antibody patients. Individual OPLS‐DA models were developed for each AE subtype, compared to the DRE group. These models achieved cross validation accuracies of 80.0 ± 5.1%, 82.3 ± 5%, and 80.4 ± 7.3% for distinguishing CASPR2‐, LGI1‐ and NMDAR‐antibody encephalitis, respectively, from DRE (Table S1). Notably, distinct metabolite signatures were identified for each subtype, including lactate, HDL (–C**H**_3_, −(C**H**_2_)_n_−), and phosphatidylcholine for CASPR2; HDL (–C**H**_3_, −(C**H**_2_)_n_−), lactate, phosphatidylcholine, glucose and UFA for LGI1; and phosphatidylcholine, HDL (−C**H**_3_, −(C**H**_2_)_n_−), and glycoprotein A (GlycA) for NMDAR (Fig. 3A, Fig. S3).

Pairwise OPLS‐DA models were built within the three AE subtypes to further study if each subtype can be stratified based on the differences in the metabolomic alteration. The accuracies of the models (CASPR2 vs. LGI1, CASPR2 vs. NMDAR, LGI1 vs. NMDAR) were 69.2 ± 3.0%, 68.9 ± 5.4%, and 77.5 ± 5.0%, respectively (Table S1). The significantly superior performance of the models than random chance (*p* <0.001, Kolmogorov–Smirnov test) indicated distinct metabolomic alterations exist within the three AE subtypes (Figs. S4 and S5, Table S4). Specific alterations in plasma metabolome in each AE subtype relative to DRE and each other are summarized in the Venn diagram (Fig. 3B).

### Potential confounding factors including seizure semiologies

To investigate whether different seizure semiologies or the seizure proximity (Table 1) were reflected in the plasma metabolome, OPLS‐DA models were built to compare patients with focal seizures (*n* = 121) versus patients with generalized seizures (*n* = 39). However, the 10‐fold cross validation demonstrated a mean accuracy of 55.8 ± 5.9%, only marginally superior to random chance. Even when employing a subset of patients with focal aware/impaired awareness seizures (*n* = 20) matched with patients experiencing generalized tonic–clonic seizures (GTCS) (*n* = 20) in terms of age, gender, and seizure‐free days, the model yielded a mean accuracy of 57.0 ± 4.7%. Similarly, when assessing the impact of seizure proximity by stratifying patients who had seizures in less than 15 days (*n* = 79) versus those without seizures for more than 300 days (*n* = 38), the model had a mean accuracy of 58.8 ± 6.0%. These results suggest that the impact of epilepsy on the blood metabolome is independent of the location, the type, and the proximity of seizure.

Other potential confounders lie in the observation that DRE and AE cohorts have multiple differences, as outlined in Table 1. To examine the influence of these potential confounders in our model, scores plots demonstrating the separation of the two groups were colored according to each variable to test for observable correlations (Fig. S6). Among these, age, the use of steroids and other immunotherapies displayed notable correlations. Consequently, we constructed OPLS‐DA models based on younger (<25, *n* = 34) versus older (>50, *n* = 32) DRE patients, and the model was able to distinguish younger versus older DRE with a 71.9 ± 4.0% cross validation accuracy. Nonetheless, the discriminatory metabolite resonances responsible for the age separation were mainly very‐low‐density lipoprotein –(C**H**_2_)_n_– (VLDL, 1.26–1.32 ppm) and unsaturated fatty acids –**H**C=C**H**– (5.25–5.38 ppm), different from those driving the separation between DRE and AE (Fig. S7).

A substantial proportion of the AE cohort was undergoing treatment with steroids and/or other immunotherapies. The OPLS‐DA model was able to distinguish between AE patients who were using steroids (*n* = 30) and those who were not (*n* = 42), with a cross validation accuracy of 65.4 ± 4.1%. AE patients on steroids exhibited elevated glucose and GlycA levels (Fig. S8). However, the OPLS‐DA model yielded only a 55.7 ± 4.5% cross validation accuracy to identify AE patients receiving other immunotherapies (*n* = 44 + 25) (Fig. S9). Therefore, while steroid administration may contribute marginally to the elevation of glucose levels in the AE versus DRE cohorts, the AE pathology remains the primary factor distinguishing their plasma metabolomics.

## Discussion

In this study, we demonstrated the ability of metabolomics to differentiate patients with AE from those with DRE, and to separate three common subtypes of autoantibody‐mediated AE. To our knowledge, this represents the first biomarker offering these discriminatory properties. While autoantibody assays will likely remain the gold standards, our NMR‐based blood test offers a promising adjunct to facilitate the diagnosis of AE given the speed of testing, affordability, and high diagnostic accuracy. Metabolomic testing may be especially valuable when patients present with seizures in the absence of obvious causes such as traumatic brain injury, neoplasms, and infectious disease. Moreover, as autoantibody assays only detect known antibodies, it is conceivable that patients harboring unknown NSAbs may be detected with NMR approaches.^26^, ^27^ While prior research has explored non‐antibody‐based biomarkers such as neurofilament light chain (NfL) and cytokines,^28^, ^29^, ^30^, ^31^ these have limitations, such as NfL's susceptibility to age and various confounding factors. Hence, the unique advantages offered by our NMR metabolomics methods in AE diagnosis and subtype differentiation may prove valuable for several applications.

In this study, we have found that different AE subtypes (CASPR2, LGI1, NMDAR) have both overlapping and distinct metabolome perturbations, suggesting the existence of both shared and distinct pathogenic mechanisms. Here we show that the common plasma metabolomic signatures shared by AE patients include decreased levels of HDL (fatty acyl chain –(C**H**_2_)_n_–, –CH_3_ resonances), phosphatidylcholine and albumin (lysyl moiety). While the clinical signs in AE are largely associated with the interaction with their respective target antigens in the CNS, there is also some peripheral expression of these proteins (e.g. LGI1), where autoimmune response might have contributed to the altered blood chemistry profiles that we have observed.^32^

Lipid profiles, especially with decreased HDL levels, are implicated in inflammatory and autoimmune diseases. For example, low HDL cholesterol and high triglycerides levels have been associated with higher levels of multiple sclerosis disability, as well as poor recovery and relapse in NMOSD.^33^, ^34^, ^35^, ^36^ Additionally, several studies have found lower HDL‐cholesterol levels in individuals with NMDAR‐ antibody encephalitis compared to healthy controls, and associated with a poorer prognosis and increased likelihood of relapse.^37^, ^38^, ^39^

Decreased levels of –N(CH_3_)_3_ resonances from phosphatidylcholine were found in AE plasma in our study, and were highly positively correlated with HDL –CH_3_ levels (*r* = 0.95, *p* <0.001, Fig. S10). As phosphatidylcholine is the main phospholipid present in plasma and an integral component of lipoproteins (particularly HDL) the observed decrease in phosphatidylcholine levels may be attributed to the reduced levels of HDL. Additionally, the decreased levels of phosphatidylcholine may occur secondary to AE‐induced inflammation, as cellular lipid profiles are modulated following inflammatory stress, including a decrease in phosphatidylcholines.^40^

Consistent with our findings, significantly lower albumin levels have been reported in AE patients, with plasma albumin levels decreased in NMDAR‐antibody encephalitis relative to healthy controls, and pre‐treatment low plasma albumin associated with worse prognosis in AE.^41^, ^42^ Albumin is a negative acute‐phase reactant and reduced serum albumin levels have been shown to correlate with systemic and central inflammatory disease, which could be due to increased albumin degradation caused by a high catabolic rate and elevated albumin transudation resulting from increased capillary permeability.^43^, ^44^ Thus, taken together, the significant decreases observed in lipoprotein and albumin resonances of AE patients observed here, are consistent with an inflammatory metabolic signature.

Our study has also demonstrated that various subtypes of AE exhibit distinct metabolic changes, aligning with the observation that different NSAbs are often associated with distinct clinical syndromes and prognoses.^12^ Elevated lactate levels were observed in both CASPR2‐ and LGI1‐antibody patients, especially for CASPR2‐antibody patients, while elevated plasma glucose levels were found in CASPR2‐antibody AE only. GlycA levels were higher in NMDAR‐ and CASPR2‐antibody patients but lower in LGI1‐antibody patients, while UFA levels were decreased in LGI1‐antibody encephalitis only.

Lactate is one of the most enriched by‐products of cellular metabolism in tissues with immune cell infiltration. Studies have indicated that the activation of inflammatory immune cells can cause a shift from oxidative phosphorylation to aerobic glycolysis, resulting in an increase in lactate.^45^ For example, elevated levels of serum lactate, have been reported in individuals with multiple sclerosis and the increases are positively correlated with increasing disability.^46^, ^47^

GlycA/B, NMR specific biomarkers of systemic inflammation, derive from the glycan moieties of acute‐phase proteins.^48^ Studies have reported elevated levels of GlycA in patients with autoimmune diseases like rheumatoid arthritis and systemic lupus erythematosus.^49^, ^50^ Therefore, the increased GlycA levels observed in NMDAR‐antibody patients are potentially indicative of ongoing inflammatory processes in this patient population. However, alone, GlycA is a non‐specific marker.^48^

We acknowledge the limitations of our study, as it did not include healthy controls nor patients with other antibody‐mediated diseases. Consequently, it is challenging to assert whether the identified pattern is specific to AE. Nonetheless, we conducted a comparative analysis with our prior research, wherein NMR metabolomics enabled successful stratification of antibody‐positive NMOSD and relapsing remitting multiple sclerosis patients, along with the identification of an inflammatory subtype of psychosis associated with VGKC/GlyR antibody.^15^, ^16^ Notably, we observed some common signatures in the autoantibody‐positive NMOSD group, including reduced phosphatidylcholine and lactate levels, along with alterations in lipoprotein profiles.^15^ Moreover, a similar profile with reduced phosphatidylcholine and HDL levels, along with elevated glucose levels was observed in the VGKC/GlyR antibody‐positive psychosis cohort.^16^ The shared metabolic signatures in these cohorts with antibody‐mediated diseases underscore the potential relevance of lipid metabolism and glucose regulation in various autoimmune and neurological conditions, warranting further exploration of these metabolic pathways for potential biomarkers or therapeutic targets.

In conclusion, this is the first study to use NMR‐based metabolomics in distinguishing AE patients from DRE patients, highlighting the diagnostic potential of the NMR‐based blood test for such differentiation. Furthermore, each AE subtype was found to exhibit a distinct biochemical signature, providing insights into the distinct metabolic impact of the different AE target antigens. Yet, no discriminatory metabolomic signatures were observed for different seizure semiologies or proximity in the DRE cohort. However, it is clear that the blood metabolome of someone experiencing status epilepticus is significantly different from someone with control epilepsy patients.^51^ Future work need to validate identified biomarkers externally in an independent cohort. It will also be important to explore the applicability of the NMR blood test in identifying other AE subtypes, seronegative AE patients and whether the AE metabolomic signature might be used to predict the persistence of AE.

## Author Contributions

T.Y., D.C.A., S.R.I., F.P. contributed to the conception and design of the study. J.T.M.M., B.C., A.R., R.N.M., S.M., E.T., A.S., S.R.I. contributed to clinical data acquisition and interpretation. T.Y., J.T.M.M., F.P. contributed to the acquisition and analysis of NMR data. W.X., T.D., F.P. contributed to statistical analysis. W.X., D.C.A., S.R.I., F.P. contributed to drafting a significant portion of the manuscript or figures.

## Conflict of Interest Statement

SRI has received honoraria/research support from UCB, Immunovant, MedImmun, Roche, Janssen, Cerebral therapeutics, ADC therapeutics, Brain, CSL Behring, and ONO Pharma and receives licensed royalties on patent application WO/2010/046716 entitled “Neurological Autoimmune Disorder,” and has filed two other patents entitled “Diagnostic method and therapy” (WO2019211633 and US‐2021‐0071249‐A1; PCT application WO202189788A1) and “Biomarkers” (PCT/GB2022/050614 and WO202189788A1).

## Supporting information


Data S1.

## Data Availability

Anonymized data and code will be shared by request from any qualified investigator.
