# Supplementary material for: Distinct plasma metabolomic signatures differentiate autoimmune encephalitis from drug‐resistant epilepsy
Source: Ann Clin Transl Neurol. 2024 Jun 21;11(7):1897–908. doi: 10.1002/acn3.52112 (PMC11251473; doi:10.1002/acn3.52112)
Supplement: Supplementary file 1 — Data S1. [file ACN3-11-1897-s001.pdf]

Supplementary Materials

(A)

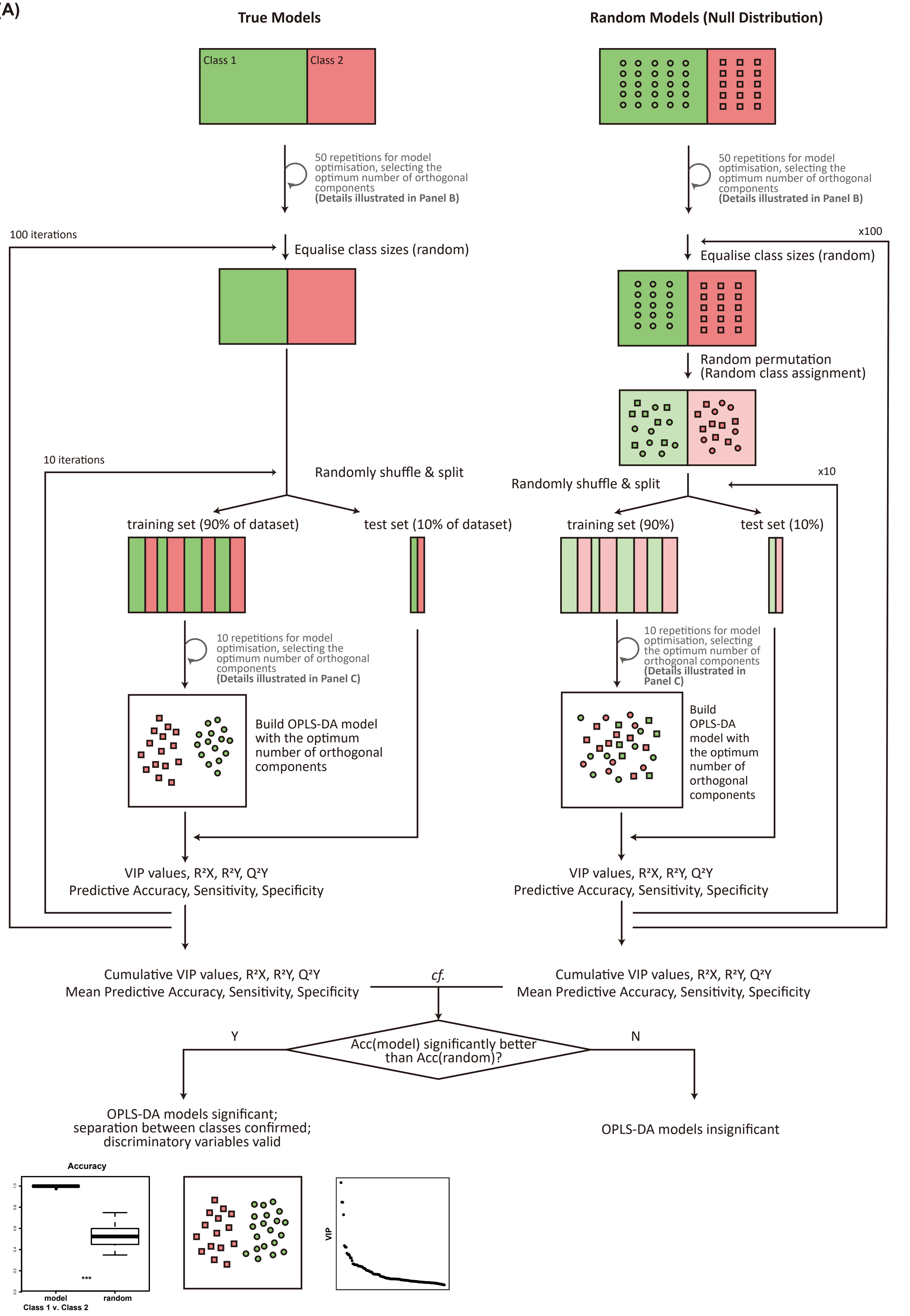

(B)

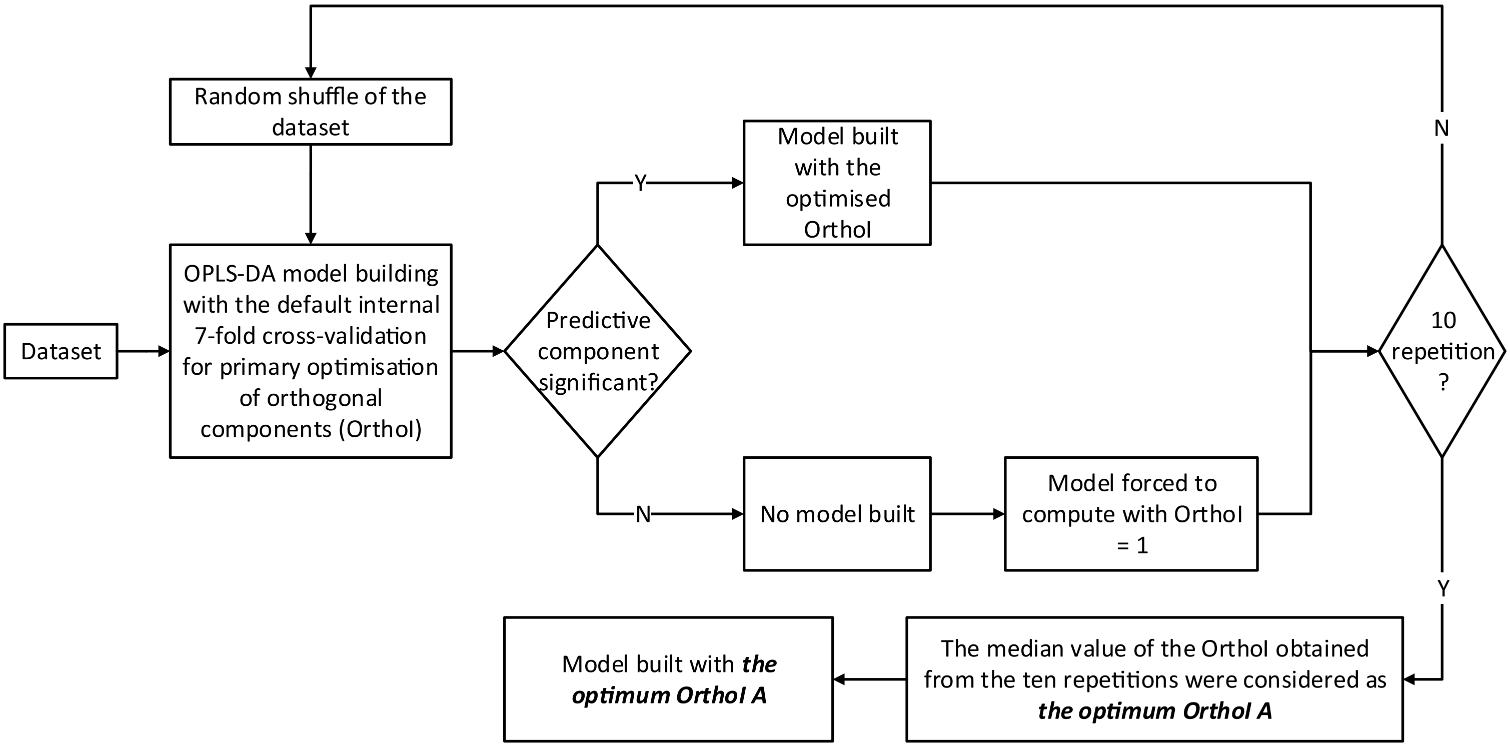

(C)

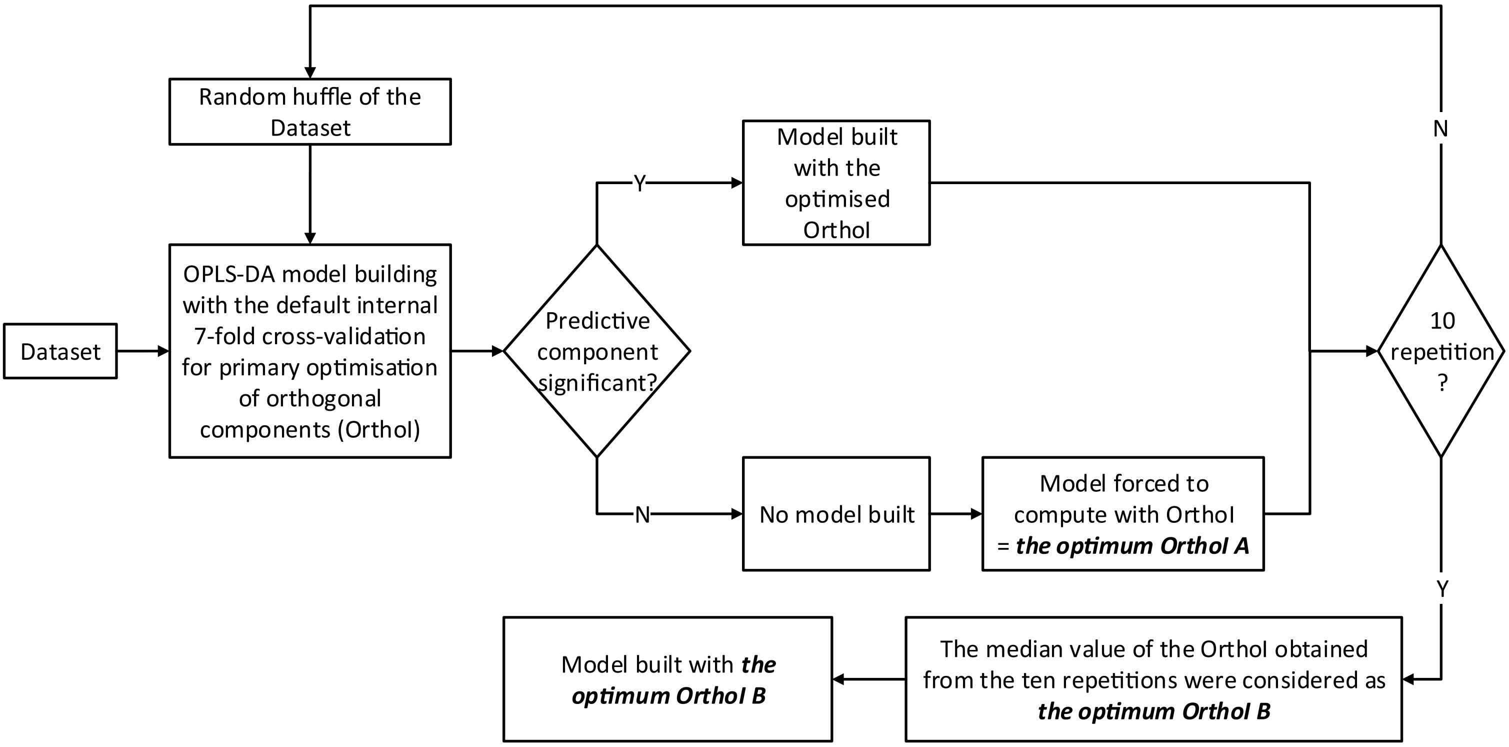

Figure S1. Schematic representation of model optimisation, cross-validation and permutation strategy

(A) details for the OPLS-DA model building and cross-validation

OPLS-DA model was built using the '*ropls*' package, employing *q2* and internal 7-fold cross-validation to optimise the number of orthogonal components (OrthoI). To address the instability of the OrthoI selected by the built-in 'ropls' package for this specific dataset, a 10-time repetition was introduced (**Panel B**). This involved shuffling the dataset and constructing OPLS-DA models ten times. The median OrthoI derived from the ten models was considered the optimum choice for building the OPLS-DA model. The optimum orthogonal number (*The optimum OrthoI A*) for the full dataset was recorded as optimum OrthoI A, and would be used for model optimisation for the training set in the cross-validation (**Panel C**).

OPLS-DA models were subjected to rigorous validation using a 10-fold external cross-validation with 100 repetitions and permutation testing. In brief, this involves shuffling the dataset and splitting into a training set (90%) and a test set (10%), with equalised class sizes. The OPLS-DA model was trained exclusively on the training set with model optimisation (as illustrated in **panel C**) and evaluated on the test set to measure accuracy, sensitivity, and specificity.

This entire validation process was repeated 100 times, resulting in 1000 models in total. In parallel, permutation testing was used to assess whether the model performed significantly better than random chance. The null distribution was generated by randomly permuting class assignments and building OPLS-DA models with the same 10-fold cross-validation with repetition scheme. The accuracy of the true models was compared to the null distribution using the Kolmogorov-Smirnov test. Models were considered significant only if their accuracies were significantly better than random chance (~50%). Discriminatory variables were identified by calculating the average of the variable importance in projection (VIP) scores of the ensemble of models, which indicated the contribution of a variable to the model.

(B) OPLS-DA Model optimisation for the full dataset. Representative scores plots presented in the manuscript was generated with the optimum model.

(C) OPLS-DA Model optimisation for the training set in the cross-validation. The *optimum Orthol A* from panel B was used for model optimisation for the training set in the cross-validation (**panel C**). The *optimum Orthol B* was considered the optimum number for the training set model.

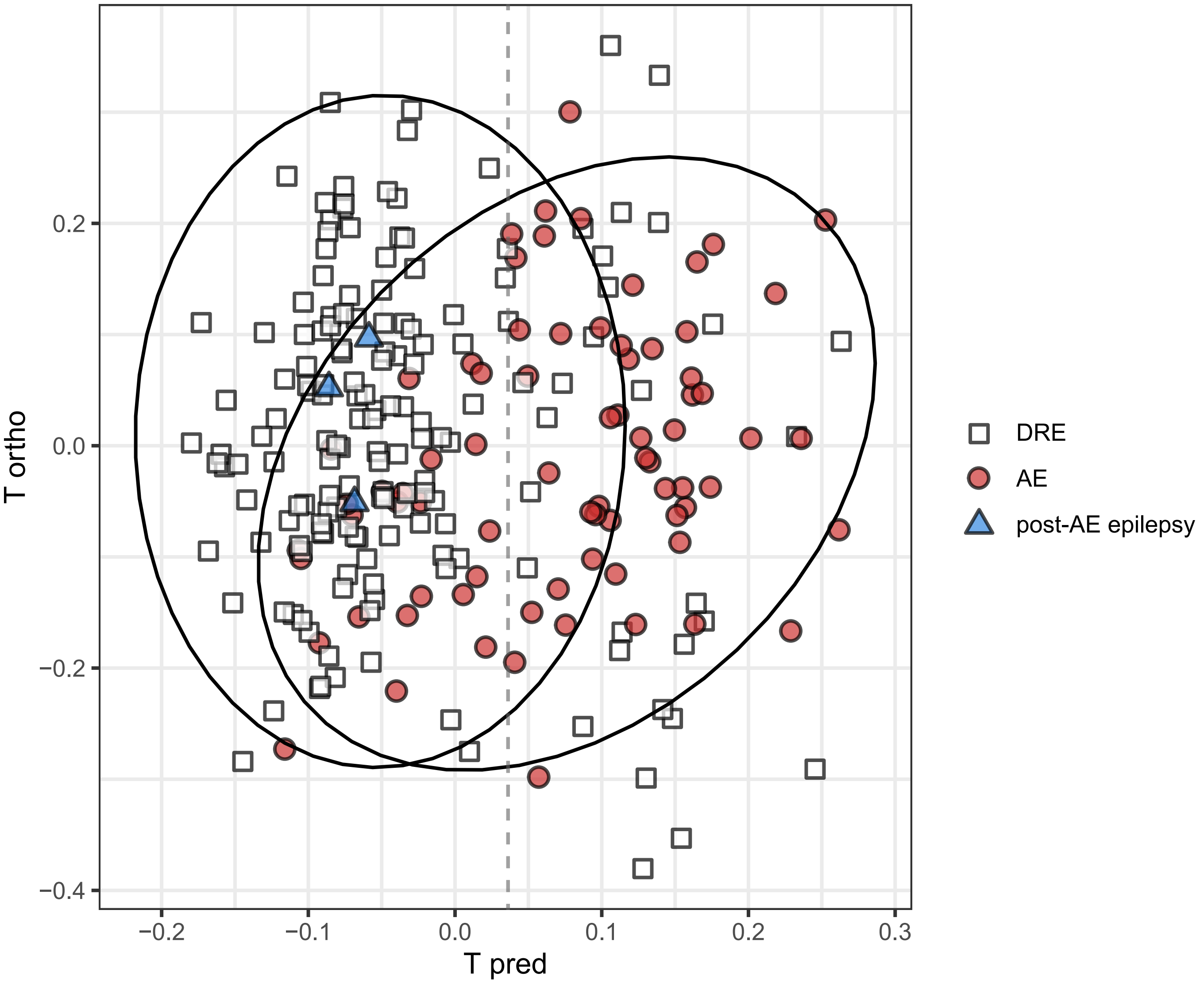

Figure S2. Prediction of subjects with post-AE epilepsy.

The OPLS-DA scores plot illustrates the application of the OPLS-DA model, differentiating AE from DRE, for the prediction of three individuals with post-AE epilepsy. The plot includes the projection of these three subjects with post-AE epilepsy onto the DRE vs AE separation. Patients situated to the left of the dashed vertical line are anticipated to have DRE.

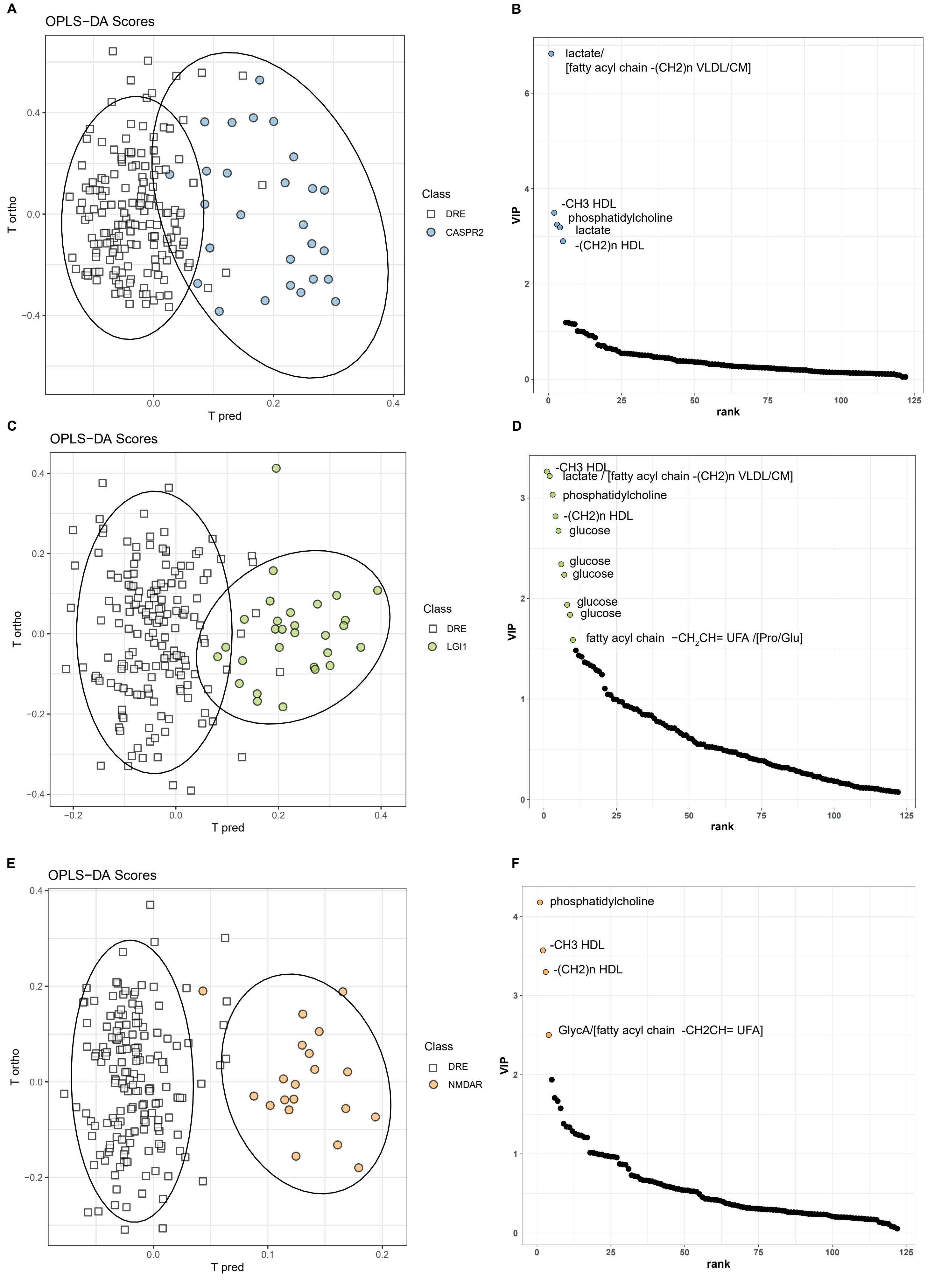

Figure S3. OPLS-DA models discriminating DRE and each AE subtype. (A, C, E) OPLS-DA scores plot differentiating between DRE and each AE subtype (CASPR2, LGI1, NMDAR). (B, D, F) Corresponding discriminatory metabolites responsible for the separation of the OPLS-DA models, ranked by VIP scores.

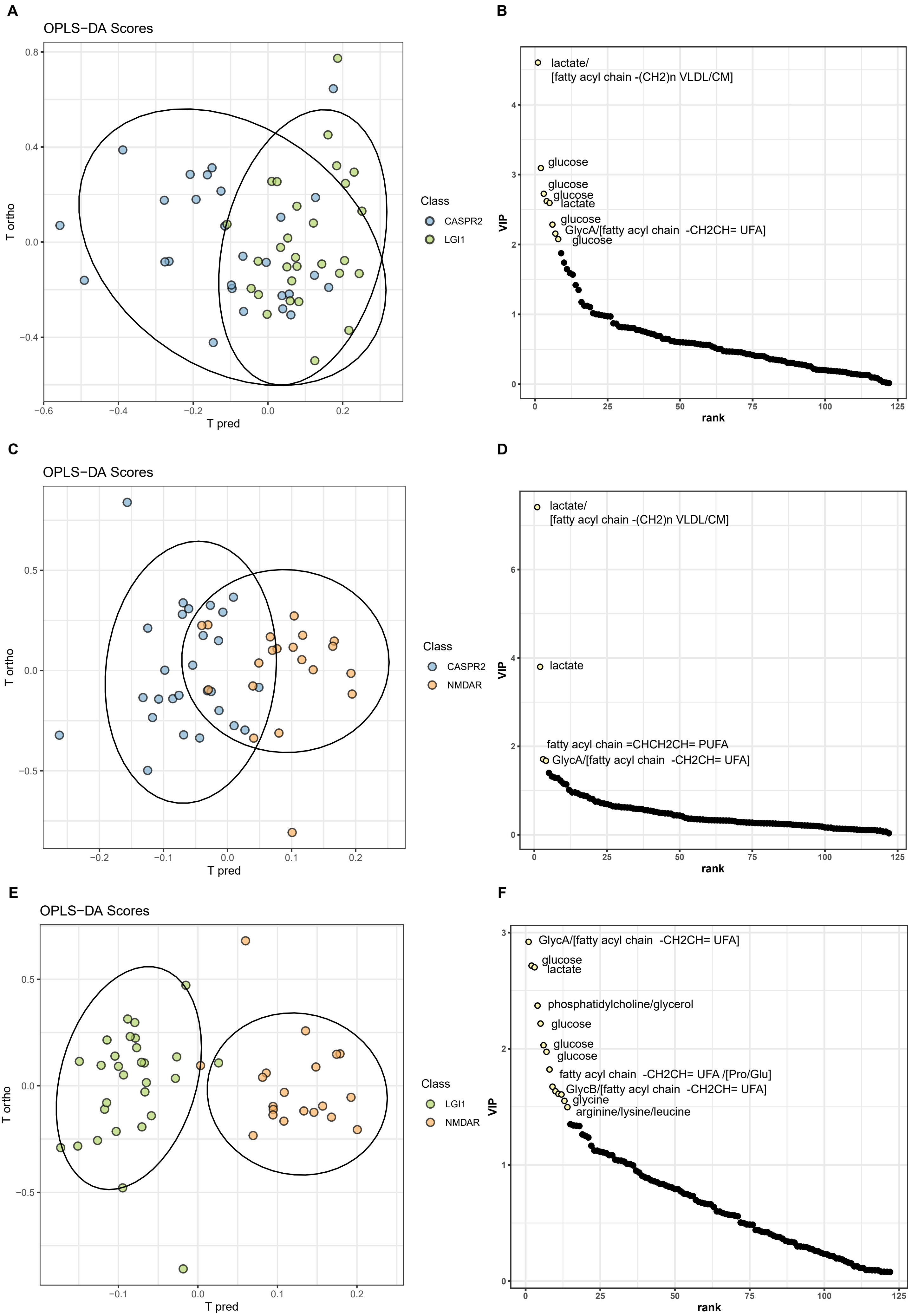

Figure S4. OPLS-DA models discriminating between AE subtypes. (A, C, E) OPLS-DA scores plot differentiating between AE subtypes (CASPR2 vs LGI1, CASPR2 vs NMDAR, LGI1 vs NMDAR). (B, D, F) Corresponding discriminatory metabolites responsible for the separation of the OPLS-DA models, ranked by VIP scores.

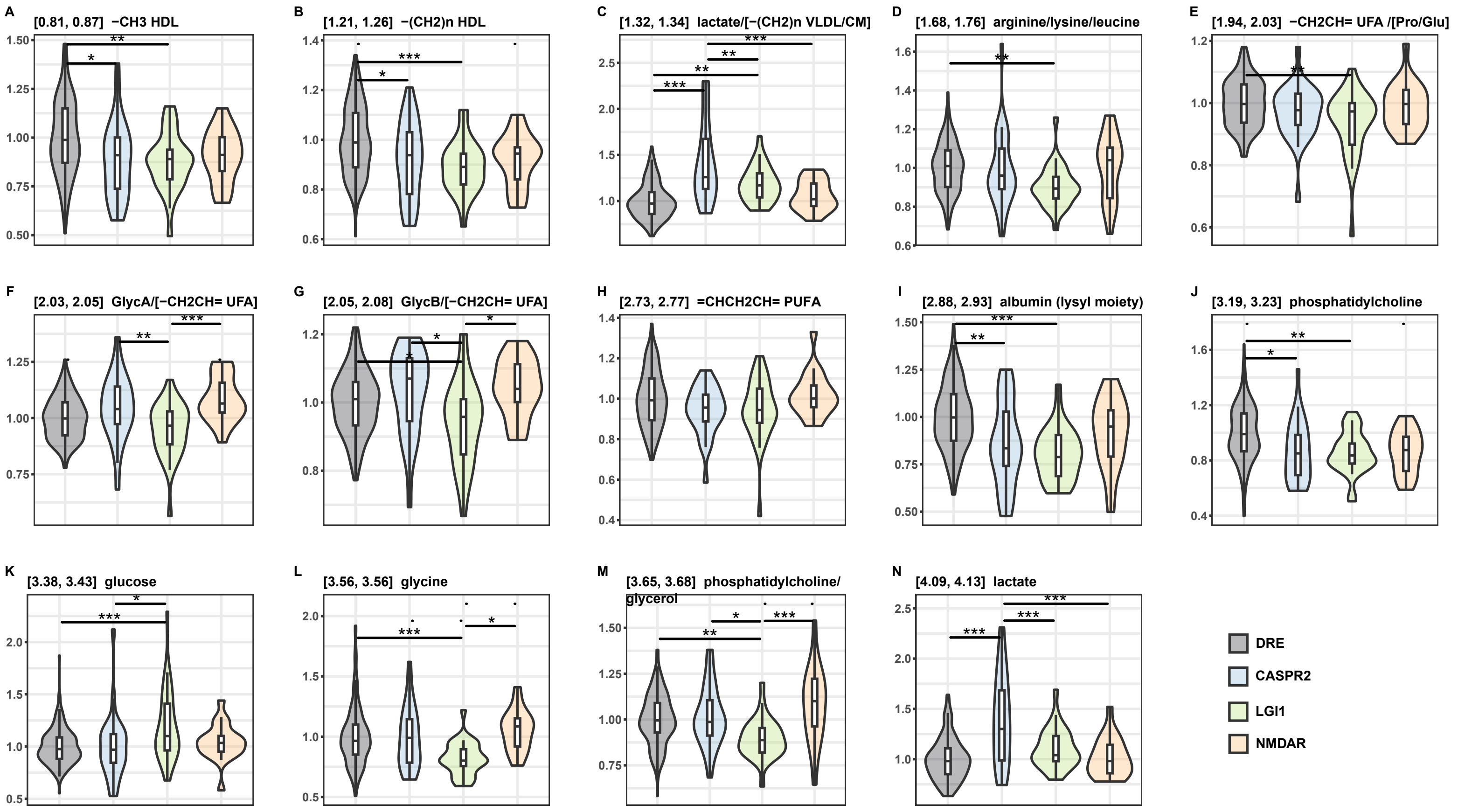

Figure S5. Violin plots of the discriminatory metabolites derived from the OPLS-DA models (AE vs DRE, CASPR2 vs LGI1, CASPR2 vs NMDAR, LGI1 vs NMDAR) in DRE (grey, n=169), CASPR2 (blue, n=27), LGI1 (green, n=30), NMDAR (orange, n=23). Significance was determined using one-way ANOVA with post hoc Tukey's HSD tests. Holm-Bonferroni method was applied for adjustment of p values due to multiple comparisons. * $q < 0.05$, ** $q < 0.01$, *** $q < 0.001$. HDL, high density lipoprotein. VLDL, very low-density lipoprotein. CM, chylomicrons. GlycA/B, glycoprotein A/B. UFA, unsaturated fatty acids. UFA, unsaturated fatty acids. PUFA, polyunsaturated fatty acids.

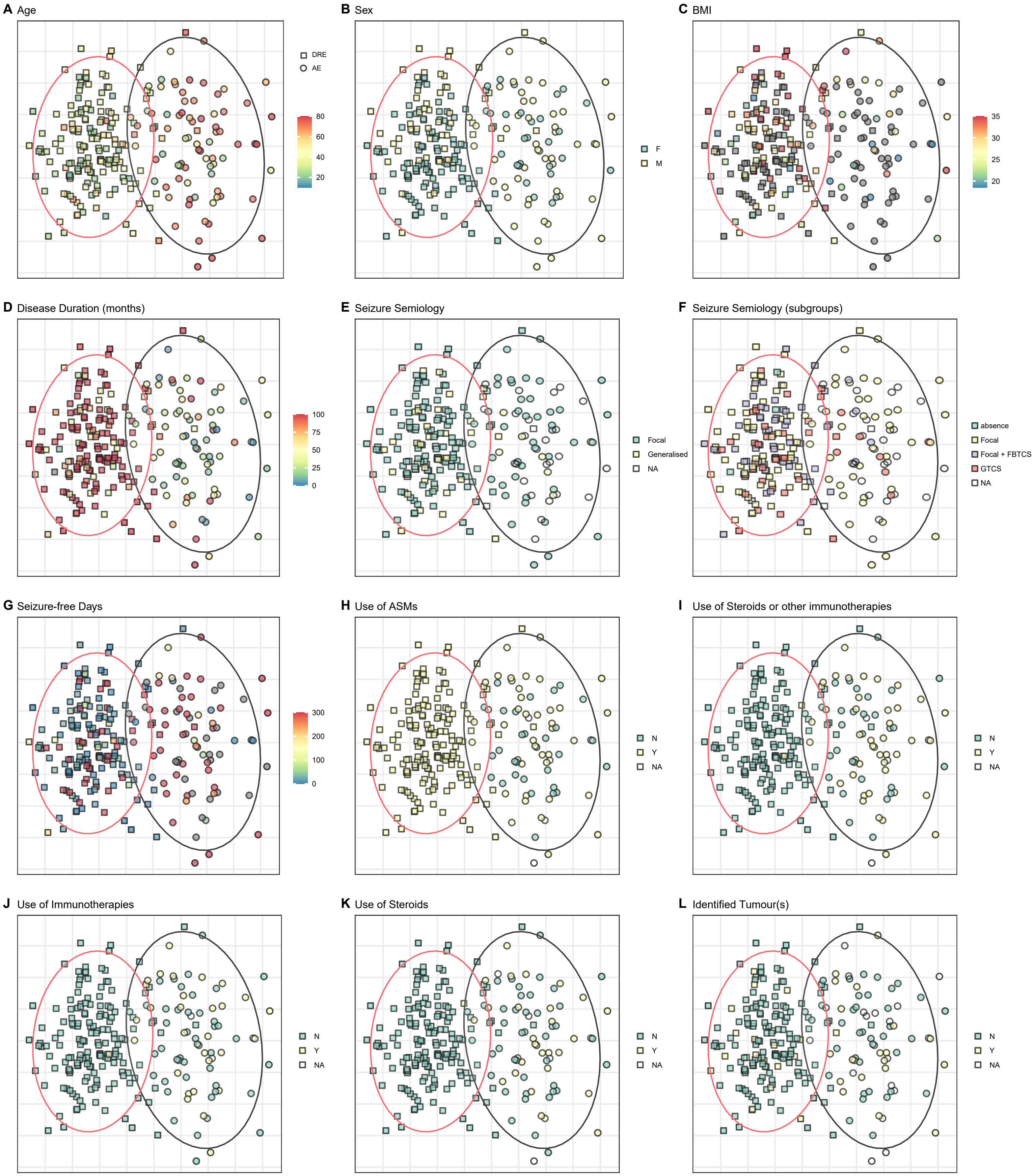

Figure S6. OPLS-DA scores plot of AE (circle) vs DRE (square) coloured by potential confounding factors.

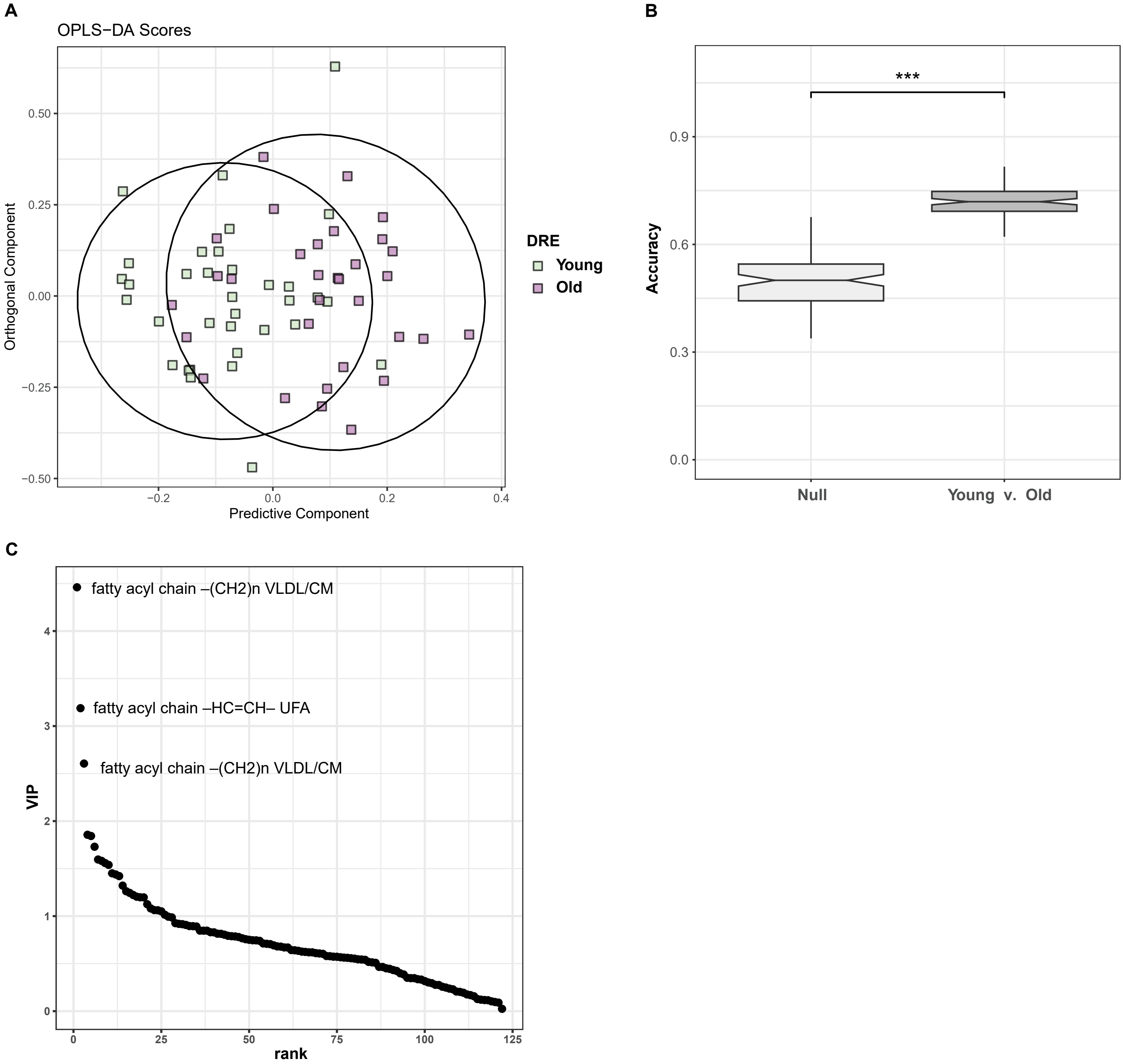

Figure S7. OPLS-DA was able to distinguish younger vs older DRE with a 71.9% cross-validation accuracy (A, B). VLDL and unsaturated fatty acids were responsible for driving the separation in age (C).

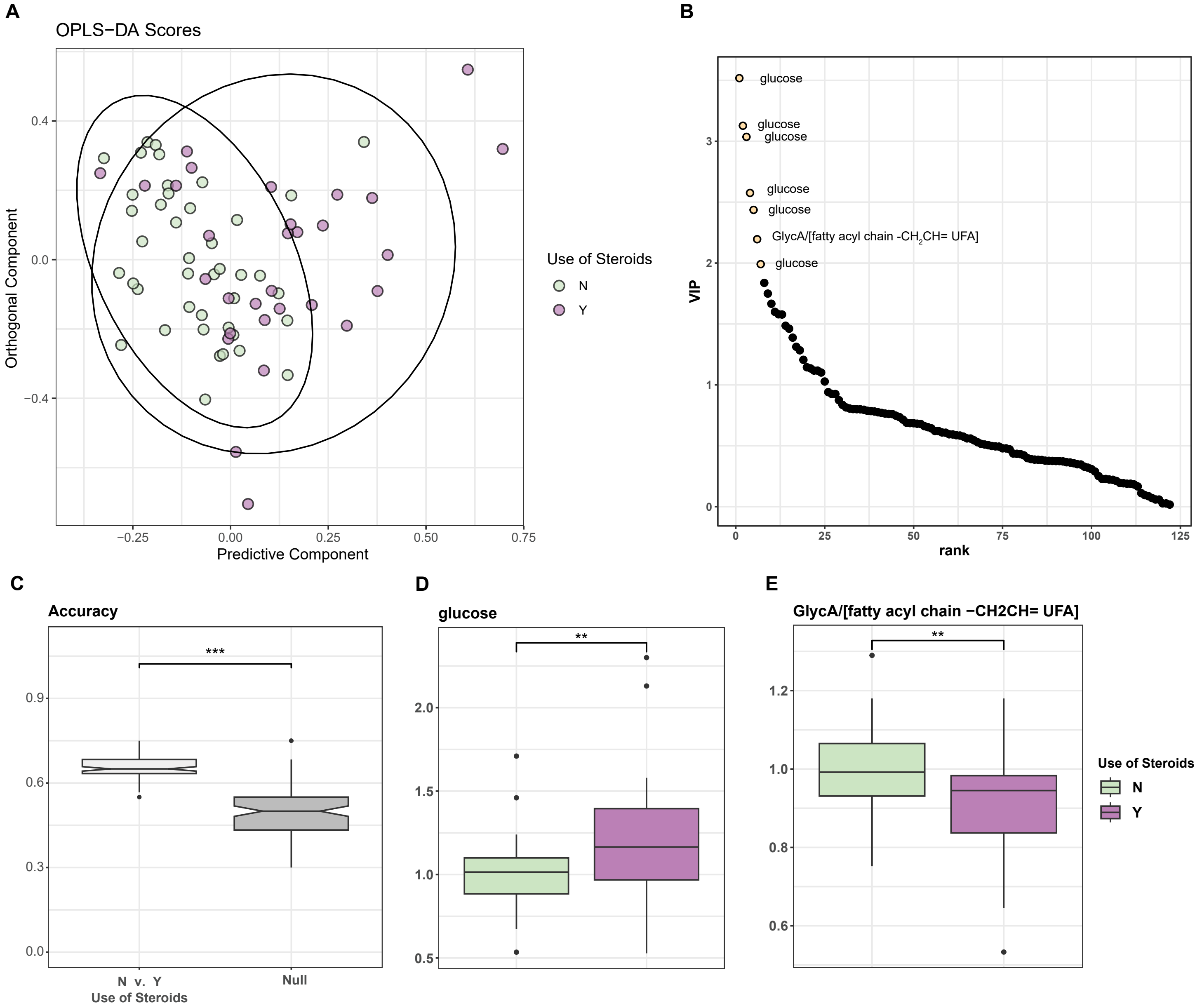

Figure S8. OPLS-DA was able to distinguish between AE patients who were using steroids and those who were not a 65.1% cross-validation accuracy (A, C). Glucose and GlycA were responsible for driving the separation in Steroid usage (B, D, E).

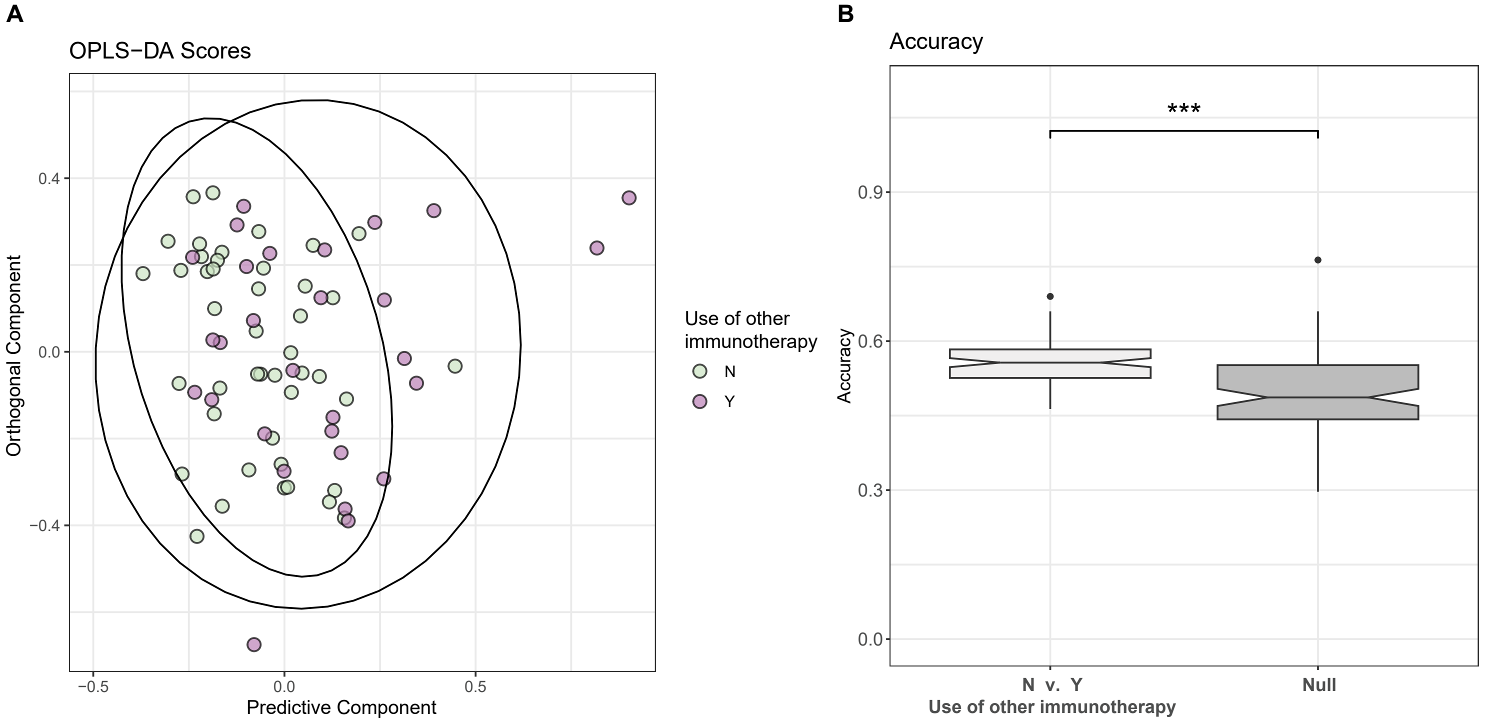

Figure S9. OPLS-DA yielded only a 55.7 ± 4.5% cross-validation accuracy to identify AE patients receiving immunotherapies (A, B)

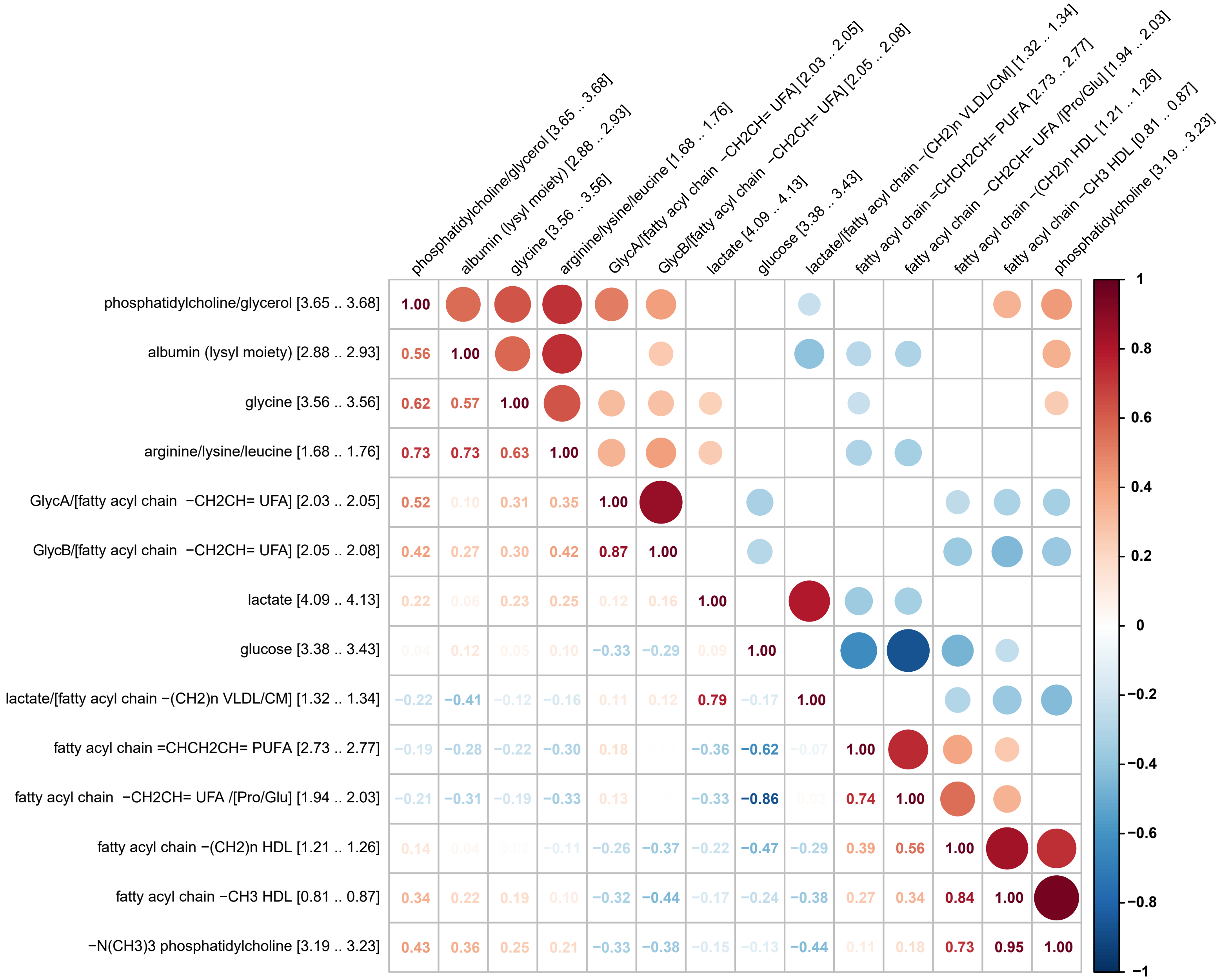

Figure S10. Correlation plot of discriminatory metabolites from the OPLS-DA models. The correlation plot encompasses all discriminatory metabolites extracted from the OPLS-DA models involving different comparisons (AE vs DRE, CASPR2 vs LGI1, CASPR2 vs NMDAR, LGI1 vs NMDAR). Pearson correlation coefficient r is visualised with different sized circles in the upper triangular of the correlation matrix, and the original r values are shown in the lower triangular. Holm-Bonferroni method was applied for adjustment of p values due to multiple comparisons. The plot includes only significant correlations.

Table S1. Statistic metrics of OPLS-DA models

comparison	model accuracy	model sensitivity	model specificity	accuracy (random)	R^2Y	R^2Y (random)	Q^2	Q^2 (random)
AE vs DRE	87.0 ± 3.1%	87.9 ± 3.4%	86.3 ± 3.6%	50.0 ± 5.3%	0.70 ± 0.06	0.29 ± 0.04	0.53 ± 0.05	-0.44 ± 0.15
CASPR2 vs DRE	80.0 ± 5.1%	83.5 ± 6.8%	78.2 ± 5.8%	50.4 ± 8.6%	0.82 ± 0.03	0.56 ± 0.06	0.34 ± 0.11	-0.84 ± 0.42
LGI1 vs DRE	82.3 ± 5.0%	82.8 ± 6.9%	82.7 ± 6.2%	50.9 ± 9.0%	0.72 ± 0.10	0.57 ± 0.06	0.46 ± 0.10	-0.84 ± 0.40
NMDAR vs DRE	80.4 ± 7.3%	80.5 ± 7.8%	82.0 ± 9.0%	50.9 ± 9.8%	0.85 ± 0.10	0.83 ± 0.06	0.42 ± 0.11	-1.32 ± 0.73
CASPR2 vs LGI1	69.2 ± 3.0%	66.7 ± 4.4%	73.3 ± 5.8%	49.4 ± 8.5%	0.34 ± 0.01	0.22 ± 0.05	0.11 ± 0.03	-0.28 ± 0.16
CASPR2 vs NMDAR	68.9 ± 5.4%	68.8 ± 7.8%	66.5 ± 7.8%	51.4 ± 10.7%	0.60 ± 0.03	0.44 ± 0.06	0.09 ± 0.10	-0.56 ± 0.32
LGI1 vs NMDAR	77.5 ± 5.0%	79.4 ± 7.8%	76.1 ± 6.8%	50.2 ± 10.2%	0.85 ± 0.02	0.64 ± 0.06	0.24 ± 0.11	-0.86 ± 0.50

Values were presented in mean ± SD. OPLS-DA models were validated on independent test data (10%) using an external 10-fold cross-validation strategy with repetition coupled with permutation testing. Accuracy, sensitivity, and specificity were calculated from the external test set to assess the robustness and predictive ability of the models. Accuracy/Sensitivity/Specificity (random) indicated the metrics calculated using the permutated dataset. R^2 and Q^2 were calculated from the OPLS-DA model built using the full data set, where R^2 provides a measure for how much variation is represented by the model and Q^2 for the goodness of prediction.

Table S2. Detailed case information of 3 post AE patients

Post AE Case 1	- *Considered to have had a probable encephalitis with seizures, headaches and fever in June 2016 and was admitted to hospital. At that time didn't have immunotherapy.*<br>- *Returned to hospital 6 weeks later where they found a Glycine receptor antibody in her CSF but not serum and gave her steroids.*<br>- *Then went to India where she had further immunotherapy (IVIG and plasma exchange).*<br>- *Later she developed language problems and was given rituximab but was allergic*<br>- *Continued to have seizures and in July 2018 Prof Sen thought she had pharmacoresistant epilepsy following meningencephalitis. The antibody was of unclear significance given it wasn't a typical syndrome. She was treated as epilepsy after that.*<br>- *Time interval: June 2016 to July 2018 = 25 months*
Post AE Case 2	- *Presented after childbirth in November 2013 with confusion, balance problems and an EEG showing encephalopathy, treated with high dose steroids and plasma exchange*<br>- *Treated as possible autoimmune encephalitis but psychiatry also thought post-partum psychosis was in the differential*<br>- *Her MRI and CSF were normal and her antibodies in March 2014 and August 2016 were negative (NMDA/AMPA/GABAb/VGKC)*<br>- *Continue to have focal and generalised seizures every few months*<br>- *Seen in epilepsy clinic in October 2015 where no further mention is made of the possible encephalitis and she is only treated with anti-seizure medications*<br>- *Time interval: November 2013 to October 2015 = 23 months*
Post AE Case 3	- *Possible encephalitis of suspected autoimmune aetiology with no antibodies in February 2014. VKGC/NMDA/AMPA/GABAb/GlyR/paraneoplastic all -ve*<br>- *However her encephalopathy was responsive to steroids*<br>- *Had IVIG in2014 which her parents though improved seizures and plasma exchange in 2014 and Feb 2015 which had an unclear benefit (initial flurry of seizures but then seizure-free for 6 weeks)*<br>- *Had rituximab in summer 2015 to allow steroid weaning and March 2016*<br>- *In October 2016 it was felt best to discontinue immunotherapy on a risk/benefit basis and from then on was only treated with anti-seizure medications*<br>- *Time interval: February 2014 to October 2016 = 32 months*

Table S3. Discriminatory metabolites identified in the OPLS-DA models of AE vs DRE with fold changes.

Spectral bin	Assignment	DRE	AE	CASPR2	LGI1	NMDAR	VIP (rank)	t test (DRE vs AE)		
								***p* value**	***q* value**	**q.sig**
**[1.32 .. 1.34]**	**lactate/[fatty acyl chain -(CH2)n- VLDL/CM]**	1	1.22	1.42	1.18	1.06	5 (1)	5.5E-08	2.5E-07	***
**[0.81 .. 0.87]**	**fatty acyl chain -CH3 HDL**	1	0.89	0.88	0.87	0.91	4 (2)	3.1E-06	5.6E-06	***
**[3.19 .. 3.23]**	**-N(CH3)3 phosphatidylcholine**	1	0.86	0.86	0.86	0.86	4 (3)	1.4E-07	4.3E-07	***
**[1.21 .. 1.26]**	**fatty acyl chain -(CH2)n-HDL**	1	0.91	0.91	0.89	0.92	3.4 (4)	4.7E-07	1.1E-06	***
**[4.09 .. 4.13]**	**lactate**	1	1.18	1.41	1.10	1.02	1.9 (5)	2.9E-05	4.4E-05	***
**[3.38 .. 3.43]**	**glucose**	1	1.08	1.00	1.20	1.04	1.8 (6)	2.4E-02	2.4E-02	*
**[1.94 .. 2.03]**	**fatty acyl chain -CH2CH= UFA /[Pro/Glu]**	1	0.96	0.97	0.94	0.99	1.5 (8)	2.3E-03	2.9E-03	**
**[2.88 .. 2.93]**	**albumin (lysyl moiety)**	1	0.86	0.86	0.80	0.91	1.5 (9)	2.1E-08	1.9E-07	***

Levels of metabolites are normalised to the mean of DRE group. VIP scores of the ensemble of models indicates the contribution of a variable to the model. Student's t test was used to identify significant differences between AE and DRE in each key metabolite. Benjamini-Hochberg method was used to control the false discovery rate at 0.05. VIP, variable importance in projection.

Table S4. Discriminatory metabolites identified in the OPLS-DA models of AE subtypes.

Spectral bin	Assignment	DRE	AE	CASPR2	LGI1	NMDAR	Model (VIP)	ANOVA (AE subtypes)			Tukey HSD (q value)		
								p value	q value	q.sig	CASPR2 vs LGI1	CASPR2 vs NMDAR	LGI1 vs NMDAR
[1.32, 1.34]	lactate/[fatty acyl chain -(CH2)n- VLDL/CM]	1	1.22	1.42	1.18	1.06	CL (1), CN (1), LN (3)	**2.5E-04**	**8.3E-04**	***	**5.4E-03**	**7.6E-06**	3.3E-01
[4.09, 4.13]	lactate	1	1.18	1.41	1.10	1.02	CL (5), CN (2)	**6.4E-05**	**6.4E-04**	***	**4.5E-04**	**7.6E-06**	7.7E-01
[3.38, 3.43]	glucose	1	1.08	1.00	1.20	1.04	CL (2), LN (2)	**3.3E-02**	**4.7E-02**	*	**1.3E-02**	1.0E+00	1.6E-01
[2.03, 2.05]	GlycA/[fatty acyl chain -CH2CH= UFA]	1	1.03	1.05	0.95	1.08	CL (7), CN (4), LN (1)	**5.8E-04**	**1.2E-03**	**	**1.1E-02**	1.0E+00	**5.7E-04**
[1.94, 2.03]	fatty acyl chain -CH2CH= UFA /[Pro/Glu]	1	0.96	0.97	0.94	0.99	LN (8)	1.4E-01	1.4E-01	n.s.	6.8E-01	1.0E+00	2.3E-01
[2.05, 2.08]	GlycB/[fatty acyl chain -CH2CH= UFA]	1	1.01	1.03	0.94	1.04	LN (9)	**5.0E-03**	**8.3E-03**	**	**2.3E-02**	1.0E+00	**1.6E-02**
[2.73, 2.77]	fatty acyl chain =CHCH2CH= PUFA	1	0.97	0.94	0.94	1.02	CN (3)	8.6E-02	9.6E-02	n.s.	1.0E+00	9.9E-01	3.3E-01
[3.56, 3.56]	Glycine	1	0.95	0.99	0.82	1.05	LN (11)	**1.5E-04**	**7.6E-04**	***	5.4E-02	1.0E+00	**1.4E-02**
[1.68, 1.76]	arginine/lysine/leucine	1	0.97	0.99	0.90	1.00	LN (13)	5.4E-02	6.8E-02	n.s.	1.6E-01	1.0E+00	1.6E-01
[3.65, 3.68]	phosphatidylcholine/glycerol	1	1.00	1.02	0.90	1.08	LN (4)	**3.3E-04**	**8.3E-04**	***	**2.8E-02**	1.0E+00	**5.7E-04**

Levels of metabolites are normalised to the mean of DRE group. CL, CN, LN refers to the pairwise AE subtype model in which the metabolite is selected as discriminatory metabolite. One-way ANOVA was used to determine the significance among AE subtypes. Benjamini-Hochberg method was used to control the false discovery rate at 0.05.
